# Heart failure and health related quality of life

**DOI:** 10.1186/1745-0179-1-19

**Published:** 2005-10-04

**Authors:** Rui Coelho, Sónia Ramos, Joana Prata, Paulo Bettencourt, António Ferreira, Mário Cerqueira-Gomes

**Affiliations:** 1 Department of Psychiatry, Hospital de S. João, Porto Medical School, Porto, Portugal; 2 Department of Psychiatry, Hospital de Magalhães Lemos, Porto, Portugal; 3 Department of Psychiatry, Centro Hospitalar de Vila Nova de Gaia, Vila Nova de Gaia, Portugal; 4 Department of Internal Medicine, Hospital de S. João, Porto, Portugal; 5 Unit of Cardiovascular Research and Development. Hospital de S. João, Porto Medical School, Porto, Portugal

**Keywords:** Congestive Heart failure, Quality of life, Prognosis

## Abstract

Quality of life is a major goal in the context of preventive and therapeutic cardiology. It is important, both as an outcome measure in clinical trials of congestive heart failure (CHF) and as a consideration in individual physicians' therapeutic decisions. In this article, quality of life concepts are reviewed, methods of measurement are explored and clinically significant changes on prognosis are discussed. There is a need for more research which is based on carefully selected measures of quality of life chosen as being of particular importance to patients and to the hypotheses being tested.

## 1. Introduction

Congestive heart failure (CHF) is a major health problem, with an increasing incidence and a gloomy prognosis, that is often accompanied by restricted physical activity and severe complaints in several areas of quality of life [1]. Despite its high prevalence, there have been few studies of the impact of CHF on quality of life, especially in the community and in the elderly patient [2], suggesting the need of epidemiology of quality of life as a part of epidemiology of "positive mental health" strictly closed with mental health. According to Stott, to the population of ageing, elderly heart failure patients who have multiple co-morbidities, quality of life, disability and cognitive impairment are the elements which matter to measure [3].

Since 1948, when the World Health Organization defined health as being not only the absence of disease and infirmity but also the presence of physical, mental and social well-being, quality of life issues have become steadily more important in health care practice and research [4].

## 2. Definition of quality of life

The concept of quality of life is not yet defined in a uniform way, lacks clarity and even creates confusion. It seems that in medicine, the term has become a bandwagon concept for all those human needs which are often neglected in a health care field increasingly dominated by technology. It is justifiable to say that it is a term describing a field of interest rather than a single variable [5].

Health status, functional status and quality of life are three concepts often used interchangeably to refer to the same domain of "health". Guyatt used the term "health related quality of life" (HRQL) because many widely valued aspects of life are not generally considered as "health-related" include income, freedom and quality of the environment [6].

As a rule, "quality of life" is used in medicine for characterising an individual patient's quality of life from his or her own subjective perspective [5]. HRQL, to Schipper and associates, can be defined as "... the functional effect of an illness and its consequent therapy upon a patient, as perceived by the patient" [6]. It has been found logical to distinguish sharply between diseases, which explain illness behaviour, and other states of health, which do not have an explanatory element but might be seen as a consequence of having one or more illnesses [7]. So, HRQL measures the *illness experience *as opposed to the *disease *[6], it defines the patient reality, his or her point of view as opposed to the reality defined by professional medical knowledge [8].

## 3. Measuring the quality of life

Measures of the quality of life in chronically ill patients provide an important source of medical information in addition to laboratory or diagnostic tests [8] and are becoming increasingly relevant to controlled clinical trials [6]. One goal of the measurement of quality of life is to have objective evaluations of how and how much the disease influences patients'life and how patients cope with it. These evaluations may be useful as a baseline and outcome measures and should provide framework to determine the impact of any change on patients'quality of life [9].

In the first phase of quality of life research in the 1970s and early 1980s, already available psychological well-being scales were used or new ones were specifically developed for this purpose. Examples are the "Affect Balance Scale" (ABS) by Bradburn (1969); the "Quality of Well-Being Scale" (QWBS) by Kaplan *et al *(1976) and the "Psychological General Well-Being Index" (PGWB) by Dupuy (1984). This particular development has connections to the "happiness research" tradition within psychology, where well-being is discussed not only in terms of absence of negative factors (such as depressed mood), but as a positive concept.

From the 1980s onwards, in addition to the assessment of well-being and satisfaction, instruments for assessing functioning in daily life were developed. This development is subsumed under the term "health status research". Well- known examples of "health status research" instruments are the Sickness Impact Profile (SIP) by Bergner *et al *(1981); the Nottingham Health Profile (NHP) by Hunt and McEwen (1980) and the Rand SF-36 Health Status Profile by Ware and Sherbourne (1992). Although these instruments do not use the term "quality of life", studies employing them are today generally regarded as belonging to health- related quality of life research. Later – in contrast to these "generic instruments" – disease specific quality of life instruments were developed [5].

General (or generic) measures attempt to provide a summary of quality of life and they can be standardized and applied widely to those with different types of illness to enable comparisons [10], but they lack the range, sensitivity and flexibility to deal with the special problems of particular illness.

Specific measures focus on problems associated with individual disease states, patients groups or areas of function [6]. Specific instruments tend to be more responsive to changes and more sensitive in discriminating the range of impairment because of their focus on the most relevant aspects of quality of life for the problems assessed. Nevertheless, using a disease specific quality of life instrument alone can miss important aspects of the impact of a disease in quality of life [10-12]. There is a need for more research, which is based on carefully selected specific measures of quality of life chosen as being of particular importance to patients and to the hypotheses being tested [13]. Some examples of specific instruments to measure quality of life in heart failure patients are: the Chronic Heart Failure Questionnaire; the Minnesota Living with Heart Failure Questionnaire; the Yale Scale; the Quality of Life Questionnaire in Severe Heart Failure and the Kansas City Cardiomyopathy Questionnaire, etc.

As there are benefits with each type of instrument, it is recommended often that both (generic and specific instruments) should be used [10,14]. However, a critical analysis of the properties of the growing range of generic and disease specific measures is necessary in order to guide and direct researchers and clinicians towards the most appropriate measures in terms of reliability, validity and sensitivity to change [10].

Instruments used in measuring quality of life must be valid (if it is really measuring what is supposed to measure); reliable (if it gives the same measurement after repeated administration in stable patients); sensitive (if it is able to reflect clinically meaningful differences in quality of life across the broad spectrum of the clinical conditions) and responsive (if it detects changes when the patients' conditions change) [11].

## 4. Domains of quality of life

Domains of quality of life refer to areas of behaviour that are measured.

The subjective domains of quality of life are: physical functioning (the capacity to perform physical tasks); occupational functioning (quality of life should focus on the ability to perform multiple essential roles and not just on return to work); perceptions about health status (health perceptions are personal beliefs and evaluations of general health status, they are the result of integration of information and feelings about health and health limitations from the self, the medical system, the family and the society); psychological functioning and social functioning. Siegrist and Junge (see Swenson and Clinch) [11] defined social health as the dimension of an individual's well-being that concerns how the individual gets along with others, how other people react to him or her, and how the person interacts with social institutions and norms.

In the domain of objectivity we can include: health status (measured by laboratory or diagnostic tests); psycophatology (CID-10/DSM-IV-TR); socio-economic status and social support (number and quality of the contacts).

Thompson *et al *(see Majani *et al*, 1999) stated that "objective measures of quality of life often bear little relationship to life satisfaction, whereas subjective indicators are often found to correlate highly with a global sense of well-being, as well as being more meaningful and sensitive barometers of quality of life". Accordingly, patients'subjective satisfaction should always be included in routine assessment and clinical interventions; they are a useful source of information on patient distress and psychological resources [15].

## 5. Assessing quality of life among people with CHF

Advanced CHF is the final outcome of many cardiovascular disorders. Despite recent improvement in survival related to newer therapies, CHF remains a condition with such a generally poor prognosis that the critical therapeutic advantages are more likely to be those that maintain and stabilize the patient's limited functional abilities and, by ameliorating symptoms, improve the comfort of the patient for the remaining duration of life. Prolongation of survival without these benefits may be viewed as a less important objective [16,17]. According to Coats *et al *optimally treated patients are often left with unrelieved symptoms and have a correspondingly low quality of life [18]. The development in the recent years of standardised measures of quality of life in CHF reflects the growing perception of the importance of these outcomes in patients [19]. The achievement of quality of life measures in CHF can be summarised as follows: general profiles of populations with CHF in terms of quality of life have been developed; the validity of the measuring instruments has been tested and correlated with clinical outcome; the need to include quality of life studies as outcome measures in clinical trials such as SOLVD has been recognised [9].

### 5.1 Quality of life in patients with CHF: comparison with other chronic diseases

Juenger *et al *compared the quality of life of patients with CHF with a previously characterised general population and with those with other chronic diseases. Patients'self assessment of quality of life was measured by the SF-36. The authors concluded that the total CHF sample was characterised by significantly reduced scores in all aspects of quality of life compared with a healthy reference group. Patients with CHF showed the same pattern of reduced quality of life as patients on chronic haemodialysis, thus it could be argued that all chronic disease conditions have a similar impact on quality of life, however patients with chronic hepatitis C had higher scores in physical functioning, role functioning, physical and general health than the CHF population. In comparison with patients with major depression from the Medical Outcome Study, the total CHF sample was characterised by significantly worse physical health (as expected) and better mental health. However, patients with more advanced CHF (NYHA III) had similar scores to patients with major depression on the mental health scales in addition to their already dramatically reduced physical health. These data are in accord with the findings of some recent studies showing that a large proportion of patients with CHF suffer from depression. [20]

### 5.2 Quality of life, medical treatment and clinical outcome

As has occurred with other chronic medical problems for which several therapies have produced comparable survival benefit, additional attributes or consequences of these therapies are then considered to warrant evaluation, among them the effects on quality of life, as a means to select an optimal drug regimen. The emergence of quality of life as an outcome measure in clinical trials of cardiovascular, as well as other therapies appears associated with additional changes in contemporary medical care [16].

Wenger suggested that quality of life measurements are particularly useful with respect to investigating treatment of cardiovascular disease in three instances: 1) when there is little likelihood of one treatment showing a major improvement in survival over another in a clinical trial. Quality of life measurement in such a trial might point toward the choice of the therapy showing the greatest benefit for improving it; 2) when a treatment is effective in reducing mortality, but has toxic or unacceptable side effects. Quality of life measurement in this case may help physicians and their patients weight the benefits and risks of such a treatment; 3) when patients are asymptomatic or have mild symptoms, the morbidity and mortality rates are low, and the therapy is long term. Quality of life measurement would ensure that quality of life is not diminished unacceptably and there is reasonable chance of compliance with therapy [11]. This is one of the areas where most of medical research is currently centred on and it is strongly supported by the pharmaceutical industry [21].

According to Konstam *et al *(1996) attention has recently been directed to quality of life in the treatment of CHF patients; however, few systematic studies actually depict the relation between quality of life and clinical outcome. These authors found that the baseline assessment of quality of life independently predicted mortality and CHF related hospitalisations in symptomatic and asymptomatic patients randomised to enalapril or placebo treatment (Studies of Left Ventricular Dysfunction trial- SOLVD – patients with an ejection fraction < 0.35 were followed for a mean of 36.5 months). The domains of activities of daily living (ADL) and general health predicted mortality and CHF related hospitalisations in both univariate and multivariate analysis. Quality of life indexes provided additional predictive values with respect to both mortality and CHF related hospitalisations, above and beyond the predictive power of variables such as ejection fraction, age, treatment (enalapril vs placebo) and the NYHA classification. These findings support the argument that a patient functional and psychological status represent independent risk factors for morbidity and mortality and warrant the need for further investigations regarding the mechanisms by which self-rated activity level predicts mortality and CHF related hospitalisations (this might include a closer look on psychological mechanisms and the pathophysiology underlying activity level). This study also supports the need to include quality of life in the evaluation and course of treatment of CHF [22].

Jenkinson *et al *(see O'Keefe *et al*, 1998) noted that the SF-36 and the Darmouth CCOP, two generic measures of quality of life were not responsive to self-reported improvements in global health in a study of elderly CHF patients starting treatment with ACE inhibitor. O'Keefe *et al *used the Chronic Heart Failure Questionnaire (CHQ), a CHF specific quality of life measure, in a similar population and found different results. It maybe possible that ACE inhibitors, despite their beneficial effect on mortality, do not lead to major improvement in quality of life; however, the better responsiveness of the CHQ may simply reflect that it is an instrument specifically designed for use in CHF [19].

Many studies have been performed in patients with CHF of various aetiologies and the beneficial effect of long-term beta blockade, has been confirmed [23]. According to these authors, numerous studies have shown that beta blockade when given for more than two months, elicit significant improvement in functional class, exercise capacity, cardiac function, quality of life and/or morbidity. Several large studies have also reported benefits on mortality and morbidity. Randomised placebo controlled trials with β_1 _blockade in patients with CHF reporting quality of life or NYHA functional class have yielded inconsistent results. While studies with β_1 _selective agents have shown improvement in quality of life or NYHA class, others with non-selective agents have shown a less clear picture and still others have failed to show significant improvement in quality of life or NYHA class [19].

### 5.3 Quality of life and somatic variables

According to Juenger *et al *few studies have investigated the relation between quality of life and clinical variables (reflecting the severity of disease) in CHF and most of these studies achieved inconclusive results. These authors studied the relation between quality of life (assessed by a generic quality of life measure:SF-36) and somatic indices (that included the assessment of New York Heart Association – NYHA, functional class, left ventricular ejection fraction, peak oxygen uptake and the distance covered during a standardised six minute walk test). They concluded that quality of life decreases as NYHA functional class worsens. The peak oxygen uptake and the six minute walk test also showed some relation to the quality of life (patients with a more severe impairment of functional capacity had, in general, significantly lower SF-36 values). In contrast, left ventricular ejection fraction showed no clear association with quality of life. These findings may also explain why beta blockade treatment in CHF has no consistent effect on the quality of life, despite a pronounced improvement in left ventricular ejection fraction, whereas the increase in peak oxygen uptake achieved by exercise training is associated with an improvement in quality of life [20].

### 5.4 Social support as a measure of clinical outcome

Numerous studies of cardiac patients (coronary heart disease patients) have reported that lack of social support and social isolation are associated with increased risk of mortality, but the study undertaken by Murberg *et al *(2000) was the first detecting an effect of social isolation (a patient's perception that he or she is no longer able to maintain the same degree of social contacts and activities with family, other relatives and friends as previously) on mortality among CHF patients. Another finding concerns the relationship between lack of intimate network support and mortality: for the CHF patients, most of them elderly, lack of social support from a spouse seems to be a more critical factor of fatal outcome than lack of social support from primary and secondary network. The authors pointed two possible explanations for that: poor network support might be associated with poor compliance to physical and medical regimens and that poor compliance may lead, in turn, to a dismal outcome; it could be also that the association between social isolation and mortality is a reflection of some underlying factors such as subjective health or hopelessness, which have been reported in several studies to be strong predictors of mortality independently of depression [24].

### 5.5 Socio-economic status and hospital readmission

Philbin *et al *noted that the socio-economic status was an independent risk factor for CHF. The principal findings of their study suggest that differences in age, sex, race, insurance, coexistent illness and location of care do not fully explain the higher frequency of readmission among low-income patients, but rather imply that other causes may exist. The authors discuss possible causes to these findings: low-income patients may have diminished access to such care (rural hospitals may be less likely to offer comprehensive programs for management of CHF); financial constraints and educational limitations, more common among low-income persons, could compromise compliance with treatment recommendations and lead to higher rate of hospitalisations; substance abuse and cigarette smoking, more common among minorities with heart failure could also play an important role [25].

### 5.6 Quality of life and depression

Investigation of the links between emotion and the development and prognosis of CHF have been the focus of much psychosomatic research of patients with cardiac disease over the last years. Depression is the most explored psychosocial factor in patients with CHF. Anxiety, however, was far less investigated. From the available data it seems that anxiety is not affecting heart failure patients to a greater extent. However, it has been shown that emotional distress prior to hospitalization was twice as common in patients with CHF when compared to other patients [26].

With respect to depression, recent studies have shown that the presence of depressive symptoms below the severity threshold for a depressive disorder is associated with elevated cardiac mortality. However, the nature of the relationship between depressive symptoms and elevated risk of cardiac mortality is unclear.

Longitudinal studies of quality of life in people at risk for heart disease may help clarify the nature of interactions between affective states, physical and social function, health perceptions and cardiac events [11].

Rumsfeld *et al *(2003) conducted a multicenter prospective cohort study of 460 outpatients with CHF with the purpose of assess whether depressive symptoms are independently associated with changes in CHF specific health status. The patients completed a baseline Medical Outcomes Study Depression Questionnaire and both a baseline and follow-up (6 ± 2 weeks) Kansas City Cardiomyopathy Questionnaire (KCCQ) were analyzed. Approximately 30% of the patients had significant depressive symptoms at baseline. Depressed patients had markedly lower KCCQ summary scores. After adjustment for potencial confounders, depressed patients were at risk for significant worsening of their CHF symptoms, physical and social function, and quality of life. The authors concluded that depressive symptoms were a strongest predictor of short-term worsening of CHF-specific health status [27]. Also Gottlieb *et al *(2004) studied the prevalence of depression in an outpatient heart failure population and its relationship to quality of life. A total of 155 patients were evaluated with the Medical Outcomes Study-Depression questionnaire, the Minnesota Living with Heart Failure questionnaire and the Beck Depression Inventory (BDI). A total of 48% of the patients scored as depressed and these scored significantly worse than non-depressed patients on all components of both the questionnaires measuring quality of life. In this study depression was observed more commonly among younger than older patients. This study is consistent with the notion that depressed CHF patients may perceive their quality of life to be lower and to underestimate their functional status. The higher incidence of depression in the young suggests that depression is due to a larger disparity between the perception of functional status and the expectation. Patients'perceptions of their health status are more important than their absolute physiological impairment in determining both degree of depression and quality of life. This may lead physicians caring for depressed CHF patients to classify them as more severely compromised and rate their NYHA functional class higher [28].

Havraneck EP *et al *(2004) tried to identify the sociodemographic and clinical factors associated with the onset of depressive symptoms in outpatients with CHF. The patients were evaluated at baseline and one year later with a Medical Outcomes Study-Depression questionnaire, a Kansas City Cardiomyopathy Questionnaire (KCCQ) and a full clinical evaluation including patients social and economic status. Of 245 patients without depressive symptoms at baseline, 52 (21,2%) developed depressive symptoms one year later. In multivariable analysis, living alone, alcohol abuse, perception of medical care as being a substancial economic burden, and health status as measured by the KCCQ were independent predictors of developing depressive symptoms. The results of this study support the use of measures of quality of life in evaluating patients with CHF. Health status measures have been shown to predict mortality and cardiac events in cardiovascular populations, including CHF. The KCCQ scores have previously been associated with subsequent mortality and hospitalization in patients with CHF. In this study, KCCQ score was one of four independent risk factors for the development of depression among outpatients with CHF, so, it may aid in identification of patients at risk for a wide range of adverse outcomes. Future studies are warranted to evaluate whether health status-guided management of patients with CHF can improve outcomes [29].

The finding of an association between psychosocial factors and morbidity and mortality in patients with CHF underscore the importance of beginning to target these factors and not just tradicional medical factors for intervention. The failure of medical therapy to produce marked improvement in quality of life is sobering and highlights the need to examine other methods of improving psychosocial outcomes in patients with CHF. Although few investigators have examined the effect of interventions on psychosocial variables, there is evidence that nonpharmacologic interventions may be quite effective in improving psychosocial outcomes. For example, exercise, CHF disease management programs, stress management and cognitive therapy, biofeedback relaxation [30], well-being therapy [31] have all been shown to improve quality of life or depression. Interventions aimed at improving social support have been successful in other populations and deserve attention in patients with CHF. In general, nonpharmacological intervention appears to be a fruitful area for future research and practice.

### 5.7 Quality of life and reabilitations programs

Quality of life is a major goal in the context of preventive and therapeutic cardiology. Randomized controlled trials have demonstrated that comprehensive cardiac rehabilitation can enhance quality of life by decreasing specific symptoms, augmenting functional capacity and enhancing mood state [32,33].

Fonarow *et al *(1997) pointed out that a comprehensive heart failure management (which included adjustments in medical therapy and intensive patient education) led to improved functional status and an 85% decrease in the hospital admission rate for transplant candidates discharged after evaluation. The potential to reduce both symptoms and costs suggests that referral to a heart failure program may be appropriate not only for potential heart transplantation, but also for medical management of persistent functional class III and IV heart failure [34].

There is little information concerning the impact of therapeutic exercise programs on quality of life of patients with CHF, but benefit is likely from an earlier return to work (a gain in physical function) and an increase in aerobic power (physical function, physical role, fatigue and vitality) [35].

Wielenga *et al *(1997) studied the effect of exercise training on quality of life and exercise capacity in 35 patients with mild to moderate chronic heart failure. Three measures were used to evaluate quality of life: the Heart Patients Psychological Questionnaire; the Sickness Impact Profile and the Self- Assessment of General Well-Being. With this study the authors confirmed that exercise training can be performed safely by patients with mild to moderate CHF and that after 12 weeks of physical training an increase in exercise performance was observed. These results support the concept that exercise training is an important modality to increase quality of life in CHF [1] and may improve the psychological outlook and self confidence of patients [9]. Furthermore, the observation that the increase in training level is not reflected as an increase in peak VO_2_, suggests that the increase in exercise test duration is related to a psychological improvement, rather than to a physical improvement. This is in accordance with the well-known belief that exercise duration is a motivation-dependent test parameter [1], often explains why patients are able to accomplish many more of the activities of daily living than exercise testing suggests is possible. However, there are no standard ways to assess motivation, which is influenced by many factors such as emotional state, personality, financial gain or loss, and interpersonal relationships [11].

## 6 Conclusion

In the first documented medical journal when the term "quality of life" was used (*Annals of Internal Medicine *of 1966), the author (J.R.Elkinton) quotes Francis Bacon's view that "*the office of medicine is but to tune this curious harp of man's body and reduce it to harmony*". This is a most remarkable definition of quality of life because it stresses not only "well-being" and "satisfaction" ("the harmony within a man"), but also the relationship of a person to the environment ("harmony between a man and his world") [5]. Such an approach has obvious relevance in the assessment of quality of life in patients with chronic medical illnesses (such as congestive heart failure). Although quality of life research has its roots in the social sciences, it will be fully accepted by health care practitioners only when it answers questions directly related to clinical programs and therapeutic choices. To answer these and similar questions, future research should be used to demonstrate the links among medical interventions, clinical and physiologic changes, and quality of life [4,36].

Those involved in psychosomatic research can benefit by knowledge and familiarity with quality of life measures; their use and adaptation to psychosomatic research may help foster greater awareness and comprehension of outcomes of psychosomatic investigations by colleagues from other disciplines. Some of the generic health profiles and cardiac-disease-specific quality of life measures discussed in this review would likely be most useful alongside instruments used in psychosomatic investigations of personality, hostility, depression and social isolation, which have already been shown to be relevant to patients with heart disease [11].

The future will show whether quality of life research was a fashionable and transient movement at the end of the twentieth century or a serious endeavour with profound implications for the daily practice of medicine, for outcome assessment in clinical trials and health services research, for health needs assessment of populations, and for resource allocation [5].
